# Changes in Dietary Diversity and Subsequent All-cause and Cause-specific Mortality Among Japanese Adults: The Japan Collaborative Cohort Study

**DOI:** 10.2188/jea.JE20240422

**Published:** 2025-08-05

**Authors:** Daiki Watanabe, Isao Muraki, Koutatsu Maruyama, Akiko Tamakoshi, the JACC Study Group

**Affiliations:** 1 Faculty of Sport Sciences, Waseda University, Saitama, Japan; 2 National Institute of Health and Nutrition, National Institutes of Biomedical Innovation, Health and Nutrition, Osaka, Japan; 3 Division of Public Health, Department of Social and Environmental Medicine, Graduate School of Medicine, Osaka University, Osaka, Japan; 4 Laboratory of Community Health and Nutrition, Special Course of Food and Health Science, Department of Bioscience, Graduate School of Agriculture, Ehime University, Ehime, Japan; 5 Department of Public Health, Graduate School of Medicine, Hokkaido University, Hokkaido, Japan

**Keywords:** diet quality, food frequency questionnaire, dietary intake, outcome, dose-response relationship

## Abstract

**Background:**

Poor dietary habits are a significant changeable factor contributing to negative health effects; however, the connection between variations in dietary diversity over time and mortality remains uncertain. This study aimed to evaluate the association between longitudinal changes in the dietary diversity score (DDS) and mortality in Japanese adults.

**Methods:**

This prospective study included 20,863 adults (13,144 women, 7,719 men) aged 40–79 years from the Japan Collaborative Cohort Study. The DDS was evaluated twice, once at baseline and again 5 years later, using a validated food frequency questionnaire that assessed 33 food items. Participants were classified into four groups based on mean DDS: baseline low DDS/5 years later low DDS (*n* = 7,866; Low/Low group), baseline low DDS/5 years later high DDS (*n* = 2,951; Low/High group), baseline high DDS/5 years later low DDS (*n* = 3,000; High/Low group), and baseline high DDS/5 years later high DDS (*n* = 7,046; High/High group). Survival data were collected until 2009, and hazard ratios (HRs) for mortality were calculated using a Cox proportional hazards model.

**Results:**

During a median follow-up of 14.8 years (256,277 person-years), 2,995 deaths were documented. After adjusting for confounders, participants in the High/High group had a lower HR for mortality from all causes (HR 0.82; 95% confidence interval [CI], 0.74–0.91) and cardiovascular disease (HR 0.81; 95% CI, 0.67–0.98) than those in the Low/Low group. Similar associations were observed with dairy, soy, and vegetables/fruits, but they were validated exclusively in women.

**Conclusion:**

This study showed that maintaining a higher DDS may be associated with lower mortality in women.

## INTRODUCTION

A poor-quality diet is a modifiable risk factor for adverse health outcomes, including death and non-communicable diseases.^1^^–^^3^ In 2017, the age-adjusted mortality proportion attributable to a poor diet ranged from 13% to 38% worldwide.^1^ A study comparing diet quality between 1990 and 2010 in 187 countries reported increased consumption of unhealthy foods.^4^ Therefore, establishing effective evidence on diet quality is crucial for promoting healthy dietary habits and reducing premature death.

A healthy diet is assessed in many ways, including the Mediterranean Diet and Healthy Eating Index.^3^ A previous review found that healthy dietary patterns are associated with a reduced risk of death despite differences in assessment approaches and regions.^5^ Several prospective cohort studies have reported that longitudinal diet quality changes are associated with all-cause,^6^^–^^8^ cardiovascular disease (CVD),^6^^,^^7^^,^^9^ and cancer-related^6^ deaths in adults.

Dietary diversity is a method of profiling diets based on food intake rather than nutrient intake, serving as one approach to evaluating an individual’s diet quality.^3^ In contrast to other dietary quality assessments, dietary diversity allows the evaluation of diet quality without requiring specialized technology or expertise as it simply counts the number of different food items consumed.^10^

Although many longitudinal Japanese cohort studies have indicated a relationship between diet quality and mortality,^11^^–^^13^ they often measured diet quality only at baseline, limiting their ability to assess the impact of dietary changes on mortality.^14^ If participants’ diet changed during follow-up, baseline assessments alone could not accurately evaluate this relationship. To the best of our knowledge, the association between longitudinal dietary diversity score (DDS) changes and all-cause as well as cause-specific mortality has not been thoroughly explored in large-scale cohort studies.^15^^,^^16^

Therefore, we aimed to examine the association between longitudinal DDS changes and mortality in Japanese adults, hypothesizing an inverse relationship between mortality and better DDS changes as a higher DDS is linked to a higher nutrient intake, including protein.^14^

## METHODS

### Study population

**Figure 1.**
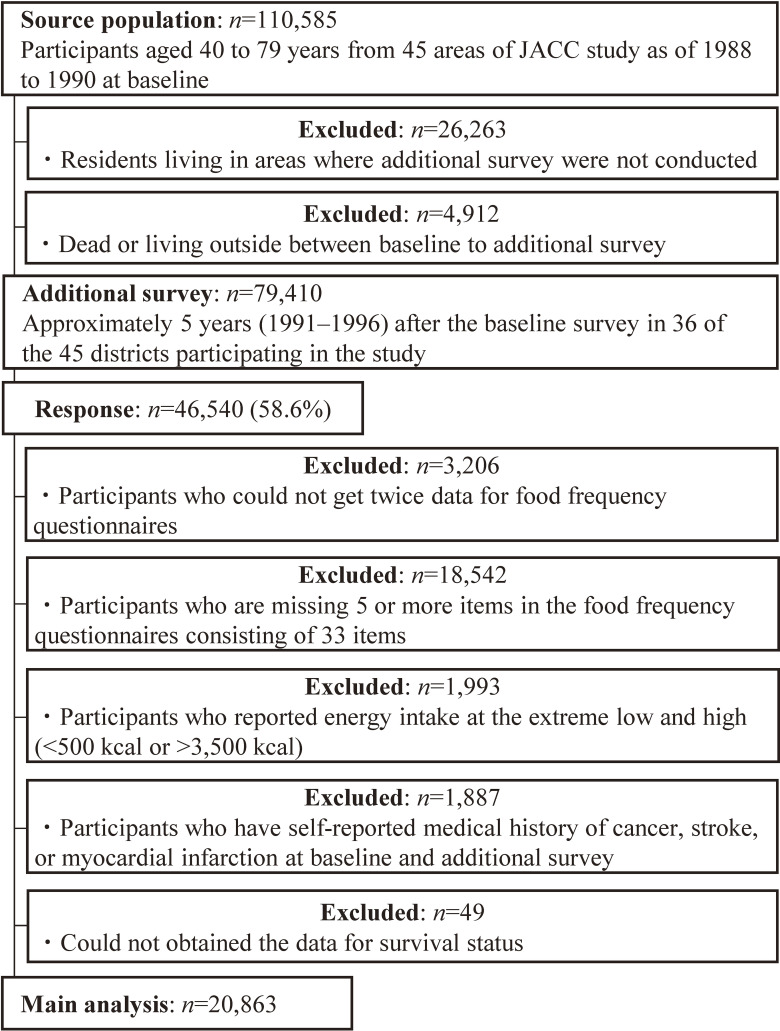
Participant flow diagram for the analysis of longitudinal changes in dietary diversity and mortality

Data were collected from Japanese adults who participated in the Japan Collaborative Cohort (JACC) Study, a large multi-site, population-based prospective cohort study. Details of this study have been previously described.^17^ Briefly, the JACC study enrolled 110,585 residents aged between 40 and 79 years from 45 areas in Japan between 1988 and 1990, with follow-up completed at the end of 2009. This study was terminated in 2009 due to the high costs involved in maintaining the cohort, and the logistical challenges of tracking participants following municipal mergers across Japan in 2000.^17^ Among the cohort members (*n* = 110,585), those who lived in areas where additional surveys were not conducted (*n* = 26,263) and those who died or moved from the study area between the baseline and additional survey (*n* = 4,912) were excluded. Of the 79,410 residents, an additional survey was conducted approximately 5 years (1991–1996) after the baseline survey in 36 of the 45 districts participating in the study (Figure 1). A total of 46,540 participants responded appropriately to the additional survey (response rate: 58.6%).

From the 46,540 residents included at baseline and in the additional surveys, we excluded individuals who: did not answer the food frequency questionnaires (FFQ) twice (*n* = 3,206) and were missing five or more items in the FFQ consisting of 33 food and beverage items (*n* = 18,542); reported extreme energy intakes (low <500 kcal or high >3,500 kcal) (*n* = 1,993); had a self-reported medical history of cancer, stroke, or myocardial infarction at baseline and/or additional survey (*n* = 1,887); and for whom survival status data could not be obtained (*n* = 49). Therefore, a total of 20,863 participants (13,144 women and 7,719 men) were included in the study.

This study was approved by the Ethics Committee of the following institutions: Hokkaido University (approval number: 14-044) and Waseda University (approval number: 2023–441). Informed consent was obtained directly from the researchers in 36 regions, whereas in nine areas, consent was obtained at the community level after the study’s purpose and data confidentiality were explained to community leaders and mayors.

### Evaluation of the DDS

The DDS and nutrient intake were assessed using a previously validated self-administered FFQ^18^ comprising 40 items, of which 33 were used to determine the DDS. These items included: potatoes, ham or sausage, liver, beef, chicken, pork (excluding ham or sausage), milk, cheese, yogurt, eggs, fresh fish, dried or salted fish, kamaboko, boiled beans, tofu, carrots or pumpkins, cabbage or head lettuce, spinach or garland chrysanthemum, tomatoes, fried vegetables, Chinese cabbage, edible wild plants, pickles, preserved foods using soy sauce, mushrooms (fungi), seaweeds (algae), citrus fruits, other fruits (excluding citrus varieties), fresh fruit juice, margarine, deep-fried foods or tempura, and sweets. Beverages, such as tea and alcohol, were excluded. Two surveys were conducted with 33 food items between 1988–1990 and 1991–1996. The DDS was calculated as described previously.^12^ Briefly, participants responded from five categories (rarely, 1–2 times/month, 1–2 times/week, 3–4 times/week, and almost every day) to questions about foods consumed in the past year. The assigned weights were 0, 0.05, 0.21, 0.5, and 1.0, respectively. The DDS was calculated by adding the weighted values based on the intake frequency of each food item,^12^ with total scores ranging from 0 (lowest dietary diversity) to 33 (highest dietary diversity). Energy and nutrient intakes were determined using the Standard Tables of Food Composition in Japan (fifth revised and enlarged edition).

Participants were divided into two groups based on their DDS (low or high DDS groups) at baseline and 5 years later according to the mean baseline (10.5 points) and 5 years later (10.3 points) DDS. Participants were then classified into the following four categories: baseline low DDS/5 years later low DDS (*n* = 7,866; Low/Low group), baseline low DDS/5 years later high DDS (*n* = 2,951; Low/High group), baseline high DDS/5 years later low DDS (*n* = 3,000; High/Low group), and baseline high DDS/5 years later high DDS (*n* = 7,046; High/High group).

### Mortality surveillance

Survival status during follow-up was confirmed by systematically reviewing the dates and causes of death from participants’ death certificates. The causes of death were coded according to the International Classification of Diseases, 10th revision (ICD-10). Cause-specific deaths were categorized into cancer (C00–C97), CVD (I01–I99), and respiratory diseases (J00–J99).^19^ If a participant in the cohort study moved out of the study area, their status was confirmed by referring to the manager of the local government office. Follow-up data were collected from the 36 areas included in the survey, with data collected until 2009.^17^

### Statistical analysis

Missing covariate data were handled by creating missing indicators. The baseline characteristics of participants from the baseline and additional surveys were compared with those included in this study.

The required sample size and survey periods for DDS evaluation based on intra- and inter- person variance at baseline and 5 years later were estimated (details in eTable 1 footnote).^14^ Adjusted dietary intake per 1,000 kcal was evaluated using the nutrient density method.^20^ The correlation between baseline DDS and dietary intake was evaluated using Spearman’s correlation analysis.

Person-years of follow-up from the return date of the additional questionnaire to the occurrence of mortality, relocation, or the end of follow-up, whichever came first, was calculated. Mortality rates for each change group are shown as deaths per 1,000 person-years. A multivariate Cox proportional hazards model was used to adjust for confounders related to DDS change group and mortality. The model’s assumptions were confirmed with the Schoenfeld residual test (*P* = 0.296). Results are presented as hazard ratios (HRs) and 95% confidence intervals (CIs), with the Low/Low group as the reference. Furthermore, the relationship between DDS change and mortality was assessed using a spline model with four knots. The HRs, with the 0-point DDS change as the reference, were calculated, and nonlinearity was tested using a Wald test.^19^^,^^21^ Additionally, building on a previous study,^12^ DDS changes for each food group and their impact on mortality risk were examined.

Sensitivity analysis was performed using three methods: 1) excluding death events recorded during the first 5 years of follow-up (325 women and 417 men),^19^ 2) conducting a similar analysis on a dataset where missing covariate values were filled in through multiple imputations,^19^ and 3) applying a uniform baseline DDS cutoff value (10.5) at baseline and 5 years later to eliminate the effect of differences in baseline and 5-year DDS cutoff values. Multiple imputation analysis was performed by pooling the results from 20 datasets generated with random numbers using the multiple imputation method to fill in the missing covariate values with the “mi estimate” command in STATA software.

The confounders for the multivariate analysis were determined based on previous studies.^6^^–^^9^^,^^15^^,^^16^^,^^19^ Model 1 was adjusted for baseline age (continuous), baseline area, and sex (women or men). Model 2 was adjusted for all the variables in model 1 and baseline body mass index (<18.5, 18.5–24.9, 25–29.9, ≥30 kg/m^2^, or missing), 5 years later smoking status (never smoked, past smoker, current smoker, or missing), 5 years later alcohol drinking (never drank, past drinker, current drinker, or missing), baseline occupation status (regular work, part-time job, self-employed, housewife, unemployed, others, or missing), baseline educational attainment (school up to age 15, 15–18, or ≥19 years, or missing), baseline marital status (married, divorced, single, widowed, or missing), baseline energy intake (continuous), baseline watching television (<2, 2–<3, 3–<4, 4–<5, ≥5 hours/day, or missing), baseline sleep duration (<6, 6–<7, 7–<8, ≥8 hours/day, or missing), baseline green tea consumption (frequency or missing), baseline coffee consumption (frequency or missing), baseline sports or exercise status (never, <1, 1–2, 3–4, ≥5 hours/week, or missing), baseline walking status (rarely, <30, 30–60, >60 minutes/day, or missing), 5 years later history of diabetes (yes, no, or missing), and baseline history of hypertension (yes, no, or missing). These covariates, including smoking and medical history, were extracted from the self-administered questionnaires at baseline and in the additional surveys.

For all analyses, a two-tailed probability of less than 5% was considered statistically significant. Statistical analyses were conducted using STATA MP, version 15.0 (StataCorp LP, College Station, TX, USA).

## RESULTS

Participants included in this study were younger, comprised a higher proportion of women, exhibited higher levels of physical activity, and had a lower prevalence of diabetes and hypertension than those who completed either or both the baseline and additional surveys (eTable 2). Table 1 shows participant characteristics according to the DDS change groups in the analyzed cohort. Participants in the High/High group were likely to be women and nonsmokers and had high physical activity levels and a low prevalence of diabetes. The mean DDS scores at baseline and 5 years later were not significantly different and moderately correlated (*r* = 0.573) (eTable 1). Group sizes required 9,309 participants to estimate a group’s “true” mean DDS change within a 95% CI with a 1% deviation.

**Table 1.** Characteristics of study participants according to longitudinal dietary diversity changes from baseline to 5 years

	Total (*n* = 20,863)		Dietary diversity score change groups							

			Low/Low (*n* = 7,866)		Low/High (*n* = 2,951)		High/Low (*n* = 3,000)		High/High (*n* = 7,046)	
Baseline age, years^a^	55.7	(9.3)	55.2	(9.6)	56.0	(9.3)	55.5	(9.4)	56.2	(8.9)
Baseline women, *n* (%)^b^	13,144	(63.0)	4,160	(52.9)	1,859	(63.0)	1,889	(63.0)	5,236	(74.3)
Baseline body mass index, kg/m^2 a^	22.7	(3.1)	22.8	(3.5)	22.7	(2.9)	22.7	(2.8)	22.7	(2.8)
Baseline current smoker, *n* (%)^b^	4,378	(21.0)	2,279	(29.0)	582	(19.7)	639	(21.3)	878	(12.5)
5 years later current smoker, *n* (%)^b^	3,925	(18.8)	1,544	(19.6)	546	(18.5)	556	(18.5)	1,279	(18.2)
Baseline current alcohol drinker, *n* (%)^b^	9,310	(44.6)	4,005	(50.9)	1,272	(43.1)	1,339	(44.6)	2,694	(38.2)
5 years later current alcohol drinker, *n* (%)^b^	8,443	(40.5)	3,195	(40.6)	1,246	(42.2)	1,179	(39.3)	2,823	(40.1)
Baseline married, *n* (%)^b^	17,856	(85.6)	6,668	(84.8)	2,520	(85.4)	2,559	(85.3)	6,109	(86.7)
Baseline no occupation, *n* (%)^b^	3,334	(16.0)	1,328	(16.9)	504	(17.1)	455	(15.2)	1,047	(14.9)
Baseline education ≥19 years, *n* (%)^b^	2,720	(13.0)	944	(12.0)	354	(12.0)	346	(11.5)	1,076	(15.3)
Baseline watching TV, hours/day^a^	2.7	(1.5)	2.7	(1.5)	2.7	(1.5)	2.7	(1.5)	2.7	(1.4)
Baseline sleep duration, hours/day^a^	7.2	(1.0)	7.2	(1.0)	7.2	(1.0)	7.2	(1.0)	7.1	(1.0)
Baseline energy intake, kcal/day^a^	1,579	(442)	1,460	(452)	1,473	(433)	1,707	(460)	1,703	(425)
Baseline green tea consumption every day, *n* (%)^b^	16,116	(77.3)	5,748	(73.1)	2,253	(76.4)	2,361	(78.7)	5,754	(81.7)
Baseline coffee consumption every day, *n* (%)^b^	10,145	(48.6)	3,670	(46.7)	1,331	(45.1)	1,462	(48.7)	3,682	(52.3)
Baseline no sports or exercise, *n* (%)^b^	15,043	(72.1)	5,952	(75.7)	2,146	(72.7)	2,175	(72.5)	4,770	(67.7)
Baseline rarely walking, *n* (%)^b^	1,994	(9.6)	890	(11.3)	293	(9.9)	297	(9.9)	514	(7.3)
Baseline diabetes, *n* (%)^b^	809	(3.9)	334	(4.3)	123	(4.2)	113	(3.8)	239	(3.4)
5 years later diabetes, *n* (%)^b^	1,086	(5.2)	423	(5.4)	149	(5.1)	165	(5.5)	349	(5.0)
Baseline hypertension, *n* (%)^b^	3,564	(17.1)	1,403	(17.8)	539	(18.3)	492	(16.4)	1,130	(16.0)
Baseline dietary diversity score^a^	10.5	(2.2)	7.4	(2.0)	8.5	(1.6)	12.7	(2.2)	14.0	(2.6)
5 years later dietary diversity score^a^	10.3	(2.1)	7.4	(1.9)	12.4	(1.9)	8.3	(1.6)	13.6	(2.5)

Four groups stratified by dietary diversity score (DDS) in baseline and 5 years later: Low/Low, low baseline DDS/low DDS 5 years later; Low/High, low baseline DDS/high DDS 5 years later; High/Low, high baseline DDS/low DDS 5 years later; High/High, high baseline DDS/high DDS 5 years later. The number of missing values for each variable is shown below: baseline body mass index (*n* = 661; 3.2%), baseline smoking status (*n* = 1,346; 6.5%), 5 years later smoking status (*n* = 1,017; 4.9%), baseline alcohol status (*n* = 591; 2.8%), 5 years later alcohol status (*n* = 1,011; 4.8%), baseline marital status (*n* = 1,031; 4.9%), baseline occupation status (*n* = 590; 2.8%), baseline education attainment (*n* = 954; 4.6%), baseline time spent watching television (*n* = 253; 1.2%), baseline sleep duration (*n* = 627; 3.0%), baseline green tea consumption (*n* = 278; 1.3%), baseline coffee consumption (*n* = 75; 0.4%), baseline sports or exercise status (*n* = 553; 2.7%), baseline walking status (*n* = 688; 3.3%), baseline history of diabetes (*n* = 1,673; 8.0%), 5 years later history of diabetes (*n* = 1,237; 5.9%), and baseline history of hypertension (*n* = 1,321; 6.3%).

^a^Continuous variables are expressed as means and standard deviations.

^b^Categorical variables are expressed as numbers and percentages.

Baseline food and nutrient intakes differed significantly between the DDS change groups. Participants in the High/High group tended to have higher protein and fat intakes (eTable 3) and greater vegetable consumption (eTable 4) than participants in other groups. In the analysis stratified by sex, the inverse correlation coefficient between DDS and carbohydrate intake tended to be stronger in women than in men (eTable 5 and eTable 6). Moreover, women tended to have higher food intakes, such as energy-adjusted intakes of fruits, vegetables, and dairy products, than men (eTable 7). However, there was no significant difference in the contribution rate of each food group to DDS between the sexes (eTable 8).

**Figure 2.**
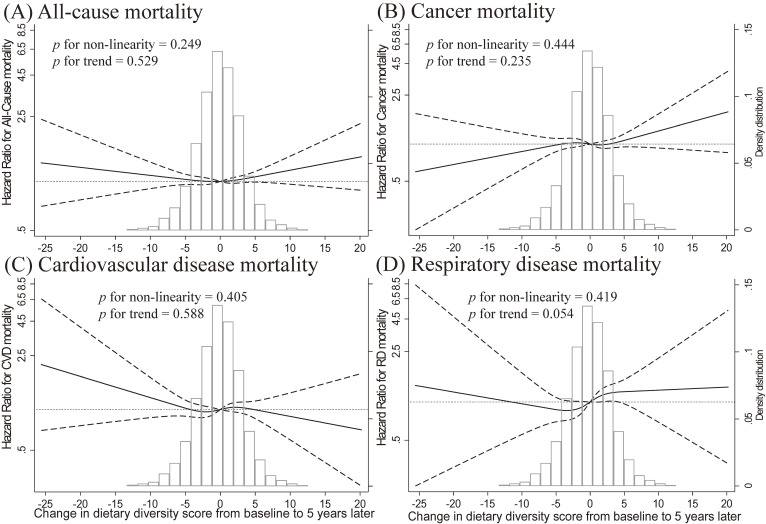
Association between change in dietary diversity score from baseline to 5 years later and all-cause and cause-specific mortality using a multivariate spline model among adults. Adjustment factors included the participants’ baseline age, baseline areas, baseline sex, baseline body mass index, 5 years later smoking status, 5 years later alcohol drinking, baseline occupation status, baseline educational attainment, baseline marital status, baseline energy intake, baseline green tea consumption, baseline coffee consumption, baseline time spent watching television, baseline sleep duration, baseline sports or exercise status, baseline walking status, 5 years later history of diabetes, and baseline history of hypertension.

Table 2 and Table 3 show the relationships between DDS change groups and mortality. The median follow-up was 14.8 (interquartile range: 6.9–16.1) years, with 2,995 deaths (14.4%) occurring during 256,277 person-years. After adjusting for confounders, participants in the High/High group had lower HRs from all-cause (HR 0.82; 95% CI, 0.74–0.91) and CVD (HR 0.81; 95% CI, 0.67–0.98) mortality than those in the Low/Low group. The sensitivity analysis yielded similar results (eTable 9, eTable 10, and eTable 11). In the subgroup analyses, a high DDS for dairy, soy, and vegetables/fruits was inversely associated with total mortality, whereas no such association was observed for meat and fish (Table 3). The high-DDS group showed lower all-cause mortality than the low-DDS group at baseline and 5 years later (eTable 12). However, changes in DDS from baseline to 5 years later were not associated with mortality in the restricted cubic spline (Figure 2) or linear models (eTable 13).

**Table 2.** Hazard ratios for longitudinal dietary diversity score change groups and all-cause and cause-specific mortality

	Dietary diversity score change groups							

	Low/Low (*n* = 7,866)		Low/High (*n* = 2,951)		High/Low (*n* = 3,000)		High/High (*n* = 7,046)	
Person years	95,143		36,616		36,982		87,536	
Baseline DDS^a^	7.4	(2.0)	8.5	(1.6)	12.7	(2.2)	14.0	(2.6)
5 years later DDS^a^	7.4	(1.9)	12.4	(1.9)	8.3	(1.6)	13.6	(2.5)
***Total mortality***								
Number of deaths	1,267		482		431		815	
Rate/1,000 PY (95% CI)	13.3	(12.6–14.1)	13.2	(12.0–14.4)	11.7	(10.6–12.8)	9.3	(8.7–10.0)
Model 1^b^	1.00	(Ref)	0.99	(0.89–1.10)	0.91	(0.82–1.02)	0.76	(0.70–0.84)
Model 2^c^	1.00	(Ref)	1.00	(0.90–1.11)	0.95	(0.85–1.06)	0.82	(0.74–0.91)
***Cancer mortality***								
Number of deaths	472		170		177		350	
Rate/1,000 PY (95% CI)	5.0	(4.5–5.4)	4.6	(4.0–5.4)	4.8	(4.1–5.5)	4.0	(3.6–4.4)
Model 1^b^	1.00	(Ref)	0.97	(0.81–1.16)	1.03	(0.86–1.22)	0.92	(0.80–1.07)
Model 2^c^	1.00	(Ref)	0.97	(0.81–1.15)	1.02	(0.85–1.22)	0.93	(0.80–1.09)
***CVD mortality***								
Number of deaths	345		134		101		213	
Rate/1,000 PY (95% CI)	3.6	(3.3–4.0)	3.7	(3.1–4.3)	2.7	(2.2–3.3)	2.4	(2.1–2.8)
Model 1^b^	1.00	(Ref)	0.98	(0.80–1.20)	0.76	(0.61–0.95)	0.70	(0.59–0.84)
Model 2^c^	1.00	(Ref)	1.00	(0.82–1.23)	0.85	(0.67–1.07)	0.81	(0.67–0.98)
***RD mortality***								
Number of deaths	135		60		50		91	
Rate/1,000 PY (95% CI)	1.4	(1.2–1.7)	1.6	(1.3–2.1)	1.4	(1.0–1.8)	1.0	(0.8–1.3)
Model 1^b^	1.00	(Ref)	1.13	(0.83–1.54)	1.03	(0.74–1.43)	0.82	(0.63–1.08)
Model 2^c^	1.00	(Ref)	1.20	(0.88–1.65)	1.14	(0.81–1.60)	0.93	(0.69–1.25)

CI, confidence interval; CVD, cardiovascular disease; DDS, dietary diversity score; PY, person-years; RD, respiratory disease; Ref, reference.

^a^Baseline dietary diversity scores are shown as means and standard deviations.

^b^Model 1: Adjusted for baseline age, baseline areas, and baseline sex.

^c^Model 2: In addition to the factors listed in model 1, adjusted for baseline body mass index, 5 years later smoking status, 5 years later alcohol drinking, baseline occupation status, baseline educational attainment, baseline marital status, baseline energy intake, baseline green tea consumption, baseline coffee consumption, baseline time spent watching television, baseline sleep duration, baseline sports or exercise status, baseline walking status, 5 years later history of diabetes, and baseline history of hypertension.

**Table 3.** Hazard ratios for longitudinal dietary diversity score change groups for each food group and all-cause mortality

	Dietary diversity score change groups							

	Low/Low		Low/High		High/Low		High/High	
**Meat, *n***	8,684		3,876		3,075		5,228	
Baseline DDS^a^	0.40	(0.22)	0.53	(0.19)	1.19	(0.40)	1.32	(0.52)
DDS in 5 years^a^	0.37	(0.19)	1.03	(0.37)	0.47	(0.17)	1.17	(0.45)
Person years	104,966		47,360		38,412		65,539	
Number of total deaths	1,397		563		419		616	
Rate/1,000 PY (95% CI)	13.3	(12.6–14.0)	11.9	(10.9–12.9)	10.9	(9.9–12.0)	9.4	(8.7–10.2)
Model 2^b^	1.00	(Ref)	1.02	(0.93–1.13)	1.02	(0.91–1.14)	1.02	(0.92–1.13)
**Fish, *n***	7,958		3,966		3,097		5,842	
Baseline DDS^a^	0.51	(0.23)	0.60	(0.22)	1.37	(0.36)	1.52	(0.43)
DDS in 5 years^a^	0.50	(0.20)	1.24	(0.34)	0.57	(0.18)	1.42	(0.42)
Person years	91,915		49,174		39,210		75,978	
Number of total deaths	1,041		586		498		870	
Rate/1,000 PY (95% CI)	11.3	(10.7–12.0)	11.9	(11.0–12.9)	12.7	(11.6–13.9)	11.5	(10.7–12.2)
Model 2^b^	1.00	(Ref)	1.00	(0.90–1.10)	1.01	(0.91–1.13)	0.95	(0.86–1.04)
**Dairy, *n***	7,198		3,283		2,322		8,060	
Baseline DDS^a^	0.19	(0.21)	0.29	(0.23)	1.16	(0.32)	1.24	(0.38)
DDS in 5 years^a^	0.24	(0.23)	1.21	(0.33)	0.37	(0.24)	1.34	(0.43)
Person years	89,996		39,951		28,961		97,370	
Number of total deaths	1,162		457		350		1,026	
Rate/1,000 PY (95% CI)	12.9	(12.2–13.7)	11.4	(10.4–12.5)	12.1	(10.9–13.4)	10.5	(9.9–11.2)
Model 2^b^	1.00	(Ref)	0.96	(0.86–1.08)	1.03	(0.91–1.16)	0.89	(0.81–0.97)
**Soy, *n***	8,736		2,322		4,510		5,295	
Baseline DDS^a^	0.35	(0.16)	0.41	(0.15)	1.01	(0.29)	1.15	(0.31)
DDS in 5 years^a^	0.43	(0.19)	1.15	(0.22)	0.51	(0.18)	1.21	(0.27)
Person years	106,658		28,467		56,388		64,765	
Number of total deaths	1,231		359		688		717	
Rate/1,000 PY (95% CI)	11.5	(10.9–12.2)	12.6	(11.4–14.0)	12.2	(11.3–13.1)	11.1	(10.3–11.9)
Model 2^b^	1.00	(Ref)	1.06	(0.90–1.11)	0.98	(0.85–1.05)	0.91	(0.83–0.99)
**Vegetables and fruits, *n***	7,018		3,226		3,649		6,970	
Baseline DDS^a^	2.41	(1.14)	2.38	(1.44)	5.21	(1.04)	5.76	(1.20)
DDS in 5 years^a^	2.64	(1.01)	5.24	(0.98)	2.93	(1.09)	5.69	(1.11)
Person years	84,990		40,335		44,463		86,489	
Number of total deaths	1,107		507		519		862	
Rate/1,000 PY (95% CI)	13.0	(12.3–13.8)	12.6	(11.5–13.7)	11.7	(10.7–12.7)	10.0	(9.3–10.7)
Model 2^b^	1.00	(Ref)	0.95	(0.85–1.06)	0.93	(0.84–1.04)	0.88	(0.80–0.97)
**Other, *n***	7,292		3,366		3,659		6,546	
Baseline DDS^a^	1.71	(0.84)	1.75	(1.02)	3.91	(0.87)	4.33	(1.05)
DDS in 5 years^a^	7.44	(0.71)	7.44	(0.77)	7.44	(0.77)	7.44	(0.94)
Person years	89,101		42,002		44,371		80,803	
Number of total deaths	1,177		538		503		777	
Rate/1,000 PY (95% CI)	13.2	(12.5–14.0)	12.8	(11.8–13.9)	11.3	(10.4–12.4)	9.6	(9.0–10.3)
Model 2^b^	1.00	(Ref)	1.00	(0.90–1.11)	0.97	(0.87–1.08)	0.90	(0.81–0.99)

CI, confidence interval; DDS, dietary diversity score; PY, person-years; Ref, reference.

The food items were categorized as follows: meat (beef, pork, ham or sausage, chicken, and liver); fish (fresh fish, kamaboko, and dried or salted fish); dairy products (milk, yogurt, and cheese); soy (boiled beans and tofu); vegetables and fruits (fried vegetables, spinach or garland chrysanthemum, carrots or pumpkins, tomatoes, cabbage or head lettuce, Chinese cabbage, edible wild plants, pickles, citrus, and non-citrus fruits).

^a^Dietary diversity scores are shown as means and standard deviations.

^b^Model 2: Adjusted for baseline age, baseline areas, baseline sex, baseline body mass index, 5 years later smoking status, 5 years later alcohol drinking, baseline occupation status, baseline educational attainment, baseline marital status, baseline energy intake, baseline green tea consumption, baseline coffee consumption, baseline time spent watching television, baseline sleep duration, baseline sports or exercise status, baseline walking status, 5 years later history of diabetes, and baseline history of hypertension.

The sex-stratified analysis showed a significant association between DDS change groups and mortality in women, with the High/High group having a lower HR for all-cause mortality than the Low/Low group (HR 0.73; 95% CI, 0.63–0.85). No such association was observed in men (HR 0.90; 95% CI, 0.78–1.03) (Table 4). There was no interaction by sex (*P* = 0.257). As information on living arrangements such as living alone was unavailable, the results were consistent when analyzed by marital status as “married” (indicating likely cohabitation) and “divorced, single, or widowed” (indicating likely living alone) (eTable 14).

**Table 4.** Hazard ratios for longitudinal dietary diversity score change groups and all-cause and cardiovascular disease mortality using a sex-stratified model

	Dietary diversity score change groups							

	Low/Low		Low/High		High/Low		High/High	
**Women, *n***	4,160		1,859		1,889		5,236	
Person years	50,312		23,471		23,011		65,603	
Baseline DDS^a^	7.6	(1.9)	8.6	(1.5)	12.7	(2.1)	14.0	(2.6)
DDS in 5 years^a^	7.6	(1.8)	12.3	(1.8)	8.5	(1.5)	13.7	(2.5)
***Total mortality***								
Number of deaths	502		205		206		421	
Rate/1,000 PY (95% CI)	10.0	(9.1–10.9)	8.7	(7.6–10.0)	9.0	(7.8–10.3)	6.4	(5.8–7.1)
Model 2^b^	1.00	(Ref)	0.87	(0.74–1.03)	0.90	(0.76–1.07)	0.73	(0.63–0.85)
***CVD mortality***								
Number of deaths	154		66		56		124	
Rate/1,000 PY (95% CI)	3.1	(2.8–3.8)	2.8	(2.2–3.6)	2.4	(2.1–3.4)	1.9	(1.7–2.4)
Model 2^b^	1.00	(Ref)	0.75	(0.42–1.31)	0.98	(0.56–1.72)	0.54	(0.31–0.94)
**Men, *n***	3,706		1,092		1,111		1,810	
Person years	44,831		13,145		13,971		21,933	
Baseline DDS^a^	7.1	(2.0)	8.3	(1.7)	12.7	(2.2)	13.8	(2.6)
DDS in 5 years^a^	7.1	(1.9)	12.4	(2.0)	8.1	(1.7)	13.5	(2.6)
***Total mortality***								
Number of deaths	765		277		225		394	
Rate/1,000 PY (95% CI)	17.1	(15.9–18.3)	21.1	(18.7–23.7)	16.1	(14.1–18.4)	18.0	(16.3–19.8)
Model 2^b^	1.00	(Ref)	1.11	(0.97–1.28)	0.99	(0.85–1.16)	0.90	(0.78–1.03)
***CVD mortality***								
Number of deaths	191		68		45		89	
Rate/1,000 PY (95% CI)	4.3	(3.7–4.9)	5.2	(4.1–6.6)	3.2	(2.4–4.3)	4.1	(3.3–5.0)
Model 2^b^	1.00	(Ref)	1.09	(0.82–1.46)	0.84	(0.60–1.18)	0.86	(0.65–1.14)

CI, confidence interval; CVD, cardiovascular disease; DDS, dietary diversity score; PY, person-years; Ref, reference.

^a^Dietary diversity scores are shown as means and standard deviations.

^b^Model 2: Adjusted for baseline age, baseline areas, baseline sex, baseline body mass index, 5 years later smoking status, 5 years later alcohol drinking, baseline occupation status, baseline educational attainment, baseline marital status, baseline energy intake, baseline green tea consumption, baseline coffee consumption, baseline time spent watching television, baseline sleep duration, baseline sports or exercise status, baseline walking status, 5 years later history of diabetes, and baseline history of hypertension.

## DISCUSSION

In this study, participants in the High/High group exhibited lower risks of all-cause and CVD mortality than those in the Low/Low group. However, these relationships were observed only in women. Moreover, changes in DDS from baseline to 5 years were not associated with mortality. To the best of our knowledge, this represents the first study to examine the association between changes in DDS and all-cause and cause-specific mortality.

Several studies have reported that maintaining a better-quality diet, calculated based on foods and nutrients, is found to be inversely related to all-cause and CVD mortality.^6^^–^^8^ However, the association between the longitudinal changes in DDS and mortality has not been fully elucidated. Two previous studies have reported that groups maintaining a high DDS are inversely associated with total mortality; however, because of the small sample size, cause-specific mortality could not be examined.^15^^,^^16^ Our study found that maintaining a high DDS was found to be inversely related to all-cause mortality and CVD risk, consistent with previous findings linking diet quality to reduced mortality risk.^15^^,^^16^ CVD deaths are attributed to poor diet quality compared with other causes of death.^1^^,^^22^ This finding is consistent with our results. Additionally, if the DDS assessments were performed only at baseline, the participants’ DDS may have changed during the follow-up period.^14^ This means that the assessment of DDS only at baseline may lead to misclassification of exposure variables. We have shown that the correlation coefficient between baseline and 5-year later DDS was 0.571, and individual DDS rankings at baseline were relatively well maintained for approximately 5 years. However, our results suggest that categorizing participants into DDS change groups based on repeated measurements may be useful for evaluating subsequent mortality risk. Notably, individuals in the High/Low group did not indicate an inverse association with death risk, unlike those in the High/High group.

Our results indicate that a change in the DDS from baseline to 5 years was not associated with mortality. In contrast, previous prospective cohort studies have reported that improvements in diet quality, as reflected in indices, such as the Mediterranean Diet score and Alternate Healthy Eating Index, are inversely associated with mortality.^6^^,^^7^^,^^9^^,^^16^ This may be because previous studies used different methods to assess dietary quality, which may have influenced the results. Although the DDS is easier to interpret than other dietary quality indices, our results, along with a previous systematic review, indicate that the DDS is not only a proxy indicator of some nutrients to be encouraged but also associated with nutrients to be discouraged, such as sodium and saturated fatty acids.^23^ This may present as a limitation when assessing dietary quality using the DDS. In this study, we could not evaluate changes in nutrient intake because the additional survey conducted between 1991 and 1996 included only 33 food items rather than all 40 food and beverage items covered in the validated FFQ. Therefore, it may be necessary to re-evaluate whether an improved DDS could help extend a healthy lifespan using a DDS evaluated by a more accurate dietary assessment method.

We found that a higher DDS was inversely related to all-cause and CVD mortality risk in women but not in men. Sex differences have been reported in the association between dietary quality and mortality risk.^8^^,^^9^ Furthermore, a previous study on Japanese adults, started in a similar era as our cohort study, found that a better-quality diet was found to be inversely related to mortality risk in women but not in men.^13^ Medical problems and diseases, such as high blood pressure and vascular disease, are more common in men, whereas dyslipidemia and inflammatory diseases are more common in women.^24^ These sex differences in medical conditions may help explain the sex differences in the association between dietary quality and mortality risk. However, excluding participants with a history of diabetes and hypertension did not fundamentally change our main results (eTable 15). Regarding dietary composition, we found that women tended to have higher energy-adjusted food intakes, including fruits, vegetables, and dairy products, than men even within the same DDS High/High group. Similar to previous studies,^25^^,^^26^ the DDS of these food groups was inversely associated with mortality risk. Therefore, the reason for the inverse association between DDS and mortality in women may be due to the higher intake of these foods in women than in men. As the sample size of men was smaller than women in this study, there may have been insufficient statistical power to detect significance between these relationships. Furthermore, these sex differences may have been influenced by factors such as cooking habits and food knowledge.^27^ A more detailed evaluation, including that of family structure (eg, living alone) and cooking habits, is needed. Therefore, future studies should collect comprehensive data on demographic factors related to sex differences and use a larger sample size to better understand the sex-specific associations between DDS changes and mortality in Japanese adults.

The detailed mechanism by which longitudinal changes in DDS are inversely related to mortality risk remains unclear; however, two possible mechanisms have been proposed in previous studies. First, dietary nutrient intake is inversely related to mortality risk. Our results indicate that participants in the High/High group, which was inversely associated with mortality, had higher protein and vitamin C intakes and lower carbohydrate intakes than those in the other groups. Previous studies have shown that the number of food items consumed is correlated with meeting the recommended nutrient intake, particularly in the Dietary Reference Intakes of older Japanese adults.^10^ This supports the idea that the High/High group, with its higher intake of a variety of food items, may have had superior nutrition compared with the other groups. Moreover, the beneficial effects of better dietary quality may also be explained by overall healthy dietary patterns and not only a specific nutrient intake.^28^^,^^29^

Second, a high DDS may help maintain a high energy intake and better health status. However, the American Heart Association suggests that increasing DDS is not an effective prevention strategy for obesity as it can be associated with higher body weight in adults.^30^ However, there are fewer obese individuals in Japan than in the United States and Europe,^31^ and it is likely that Japan, with its aging population, will experience unintended weight loss rather than obesity.^32^ A previous epidemiological study reported that DDS is found to be inversely related to all-cause and CVD mortality in middle-aged and older Japanese adults and that dietary diversity, including fruit and soy consumption, is inversely related to mortality risk—similar to our findings.^12^ Furthermore, in other countries, positive changes or long-term maintenance of dietary quality have been inversely related to mortality risk.^6^^–^^9^^,^^15^^,^^16^ These facts support our findings, which demonstrated that the High/High group with a higher DDS was found to be inversely related to all-cause and CVD mortality.

The key strengths of our study include its prospective design and the large sample size with a long follow-up period. However, this study also had a few limitations. First, we could not obtain responses to an additional survey 5 years later from all the residents who participated in the baseline survey. Participants in this study may have been more health conscious than those who did not participate (eTable 2). Second, although we used baseline and 5-year follow-up data to identify longitudinal DDS change groups, it remains uncertain whether two survey points were sufficient to fully evaluate changes in DDS over time. Although it is preferable to use DDS data with longer follow-up periods and repeated measurements, we showed that the number of measurements and sample size for the DDS used in our study were sufficient (eTable 1). Third, we evaluated exposure variables and covariates, such as body weight and smoking status, using questionnaires, which may have introduced systematic errors that affected the accurate evaluation of these variables. The accuracy of the DDS calculated using the FFQ needs to be confirmed against that calculated using more accurate methods, such as dietary records. Fourth, the dietary diversity assessment was conducted during the baseline survey from 1988 to 1990, and as dietary habits and disease patterns may have changed since then, this limits the generalizability of our findings to current Japanese dietary habits. Finally, although the study adjusted for confounders, residual confounding factors, such as economic status, may have influenced the association between DDS change and mortality. These limitations hinder the generalizability of our results.

In conclusion, our results showed that a high DDS was found to be inversely related to all-cause and CVD mortality. However, these relationships were only confirmed in women. Moreover, changes in the DDS from baseline to 5 years later were not associated with mortality, suggesting that improving DDS after middle age may not have a beneficial effect on mortality risk. These findings suggest that it may be necessary to identify and improve the diet of individuals with a poor DDS through opportunities for dietary assessment and nutritional education to encourage and maintain a high DDS in middle-aged adults, especially women. Future studies with a larger sample size and more detailed assessments may be required to further validate our findings.

## SUPPLEMENTARY MATERIAL

eTable 1. Accuracy and precision of the dietary diversity score according to sex

eTable 2. Characteristics of participants with baseline and additional surveys in the Japan Collaborative Cohort Study

eTable 3. Association between energy and nutrient intake and longitudinal dietary diversity score change groups

eTable 4. Association between food and beverage consumption and dietary diversity score change groups

eTable 5. Association between energy and nutrient intake and longitudinal dietary diversity score change groups in men

eTable 6. Association between energy and nutrient intake and longitudinal dietary diversity score change groups in women

eTable 7. Association between food and beverage consumption and dietary diversity score change groups by sex

eTable 8. Association between dietary diversity score for each food group and dietary diversity score change groups by sex

eTable 9. Sensitivity analysis results for the relationship between longitudinal longitudinal dietary diversity score change groups and all-cause and cause-specific mortality after excluding participants with an event in the first 5 years of follow-up

eTable 10. Sensitivity analysis results for the relationship between dietary diversity score change groups and all-cause and cause-specific mortality using a multiple imputation method for missing value of covariates

eTable 11. Sensitivity analysis results for the relationship between dietary diversity score change groups and all-cause and cause-specific mortality using uniform dietary diversity cutoff values at both baseline and 5 years later

eTable 12. Hazard ratios for baseline and 5 years later dietary diversity score and all-cause and cause-specific mortality calculated using a multivariate Cox proportional hazards model

eTable 13. Association between change in dietary diversity score from baseline to 5 years later and all-cause and cause-specific mortality using a multivariate linear model among adults

eTable 14. Hazard ratios for longitudinal dietary diversity score change groups and all-cause mortality calculated using sex and marital status stratified multivariate Cox proportional hazards models

eTable 15. Sensitivity analysis results for the relationship between longitudinal dietary diversity score change groups and all-cause and cardiovascular disease mortality using the sex-stratified model after excluding participants with a history of diabetes and hypertension
