# Supplementary material for: Changes in Dietary Diversity and Subsequent All-cause and Cause-specific Mortality Among Japanese Adults: The Japan Collaborative Cohort Study
Source: J Epidemiol. 2025 Aug 5;35(8):373–81. doi: 10.2188/jea.JE20240422 (PMC12237590; doi:10.2188/jea.JE20240422)
Supplement: Supplementary file 1 [file je-35-373-s001.pdf]

eTable 1. Accuracy and precision of the dietary diversity score according to sex

	Total (n=20,863) [41,726 measurements]	Men (n=7,719) [15,438 measurements]	Women (n=13,144) [26,288 measurements]
Mean DDS score at baseline	10.5 (3.7)	9.7 (3.7)	11.0 (3.7)
Mean DDS score in 5-years later	10.3 (3.5)	9.5 (3.5)	10.8 (3.5)
Spearman's rank correlation coefficient	0.573	0.535	0.573
CVw [%] [a]	22.8	25.7	21.3
CVb [%] [a]	43.6	46.4	40.8
VR	0.27	0.31	0.27
ICC [b]	0.66	0.64	0.66
Required group size [c]			
Specified % deviation			
0.5	37,235	43,327	32,534
1	9,309	10,832	8,133
2.5	1,489	1,733	1,301
5	372	433	325
Required survey periods [d]			
Specified correlation coefficient			
0.80	1	1	1
0.85	1	1	1
0.90	2	2	2
0.95	3	3	3
Required survey periods [e]			
Specified % deviation			
5	80	102	70
10	20	25	17
20	5	6	4
30	2	3	2

CI, confidence interval; CV_w, coefficient of within-person variation; CV_b, coefficient of between-person variation; DDS, dietary diversity score; ICC, intraclass correlation coefficient; VR, within-person/between-person variance ratio.

[a] The CV_w and CV_b for dietary diversity score were calculated using analysis of variance.

[b] ICC = CV_b / (CV_w + CV_b). If the ICC is comparatively high, it means a larger CV_b in dietary diversity score change.

[c] The group size = $1.96^2 \times [(CV_b^2 + CV_w^2)/D_0^2]$ required to estimate a group's "true" mean dietary diversity score change within a 95% CI with a specified % deviation (D_0), where D_0 is the specified % deviation. All values are group sizes.

[d] The number of survey periods during the study period (NT_1) = $[r^2/(1 - r^2)] \times$ VR required to obtain a specified r between an individual's measured value and unmeasured usual "true" mean dietary diversity score change, where r is the specified correlation coefficient and an index of confidence related to an individual's classification or ranking within a population. All values are from survey periods during study periods.

[e] The number of survey periods during the study period (NT_2) = $(1.96 \times CV_w/D_1)^2$ required to estimate an individual's "true" mean dietary diversity score change within a 95% CI with a specified % deviation (D_1), where D_1 is a specified % deviation. All values are from survey periods during the study periods.

eTable 2. Characteristics of participants with baseline and additional surveys in the Japan Collaborative Cohort Study

	Baseline survey		Baseline and additional surveys			
	Total (n=110,585)		Total (n=46,540)		Included participants (n=20,863)	
Age, years [a]	57.8	(10.2)	57.7	(9.6)	55.7	(9.3)
Women, *n* (%) [b]	64,190	(58.0)	28,272	(60.7)	13,144	(63.0)
Body mass index, kg/m^2 [a]	22.8	(3.5)	22.8	(3.9)	22.7	(3.1)
Current smoker, *n* (%) [b]	26,510	(24.0)	10,032	(21.6)	4,378	(21.0)
Current alcohol drinker, *n* (%) [b]	47,421	(42.9)	20,897	(44.9)	9,310	(44.6)
Married, *n* (%) [b]	82,707	(74.8)	34,661	(74.5)	17,856	(85.6)
No occupation, *n* (%) [b]	19,076	(17.3)	8,560	(18.4)	3,334	(16.0)
Education ≥19 years, *n* (%) [b]	10,590	(9.6)	4,345	(9.3)	2,720	(13.0)
Watching TV, hours/day [a]	2.8	(1.6)	2.9	(1.6)	2.7	(1.5)
Sleep duration, hours/day [a]	7.3	(1.1)	7.2	(1.1)	7.2	(1.0)
No sports or exercise, *n* (%) [b]	64,401	(58.2)	24,815	(53.3)	15,043	(72.1)
Rarely walking, *n* (%) [b]	9,685	(8.8)	3,155	(6.8)	1,994	(9.6)
Diabetes, *n* (%) [b]	5,283	(4.8)	2,283	(4.9)	809	(3.9)
Hypertension, *n* (%) [b]	22,531	(20.4)	9,101	(19.6)	3,564	(17.1)

The number of missing values for each variable is shown below (n = [n in baseline survey] and [n in additional survey]): body mass index (n=6,604 and 2,687), smoking status (n=10,960 and 5,396), alcohol status (n=8,138 and 3,557), marital status (n=15,626 and 6,954), occupation status (n=17,567 and 7,210), education attainment (n=29,306 and 11,200), time spent watching television (n=19,493 and 12,181), sleep duration (n=6,509 and 3,332), sports or exercise status (n=22,315 and 12,768), walking status (n=26,559 and 13,518), history of diabetes (n=14,504 and 6,674), and history of hypertension (n=11,806 and 5,429). Body mass index was calculated as body weight (kg) divided by height squared (m^2).

[a] Continuous variables are expressed as means and standard deviations.

[b] Categorical variables are expressed as numbers and percentages.

eTable 3. Association between energy and nutrient intake and longitudinal dietary diversity score change groups

	Total (n=20,863)	Groups of dietary diversity score change				Baseline DDS
		Low/Low (n=7,866)	Low/High (n=2,951)	High/Low (n=3,000)	High/High (n=7,046)	*r* [a]
Energy intake, kcal/day	1,517	1,382	1,392	1,619	1,624	0.34
Protein, % energy/day	13.9	12.5	13.5	14.4	15.2	0.53
Fat, % energy/day	19.1	16.3	18.0	20.0	21.7	0.51
Carbohydrate, % energy/day	61.5	63.7	63.1	60.7	59.9	-0.25
SFA, % energy/day	5.8	4.9	5.4	6.1	6.7	0.44
MUFA, % energy/day	6.1	5.1	5.7	6.4	7.0	0.50
PUFA, % energy/day	4.4	3.8	4.3	4.6	5.0	0.44
n-6 PUFA, % energy/day	3.6	3.1	3.5	3.7	4.0	0.41
n-3 PUFA, % energy/day	0.9	0.7	0.8	0.9	1.0	0.45
Cholesterol, mg/1,000 kcal/day	146	119	135	157	174	0.46
Water intake, g/1,000 kcal/day	838	780	826	843	894	0.20
Vitamin A, μg RE/1,000 kcal/day [b]	364	289	333	394	436	0.39
Vitamin D, μg/1,000 kcal/day	4.1	3.3	3.7	4.5	4.9	0.42
α-tocopherol, mg/1,000 kcal/day	3.3	2.8	3.1	3.5	3.7	0.59
Vitamin K, μg/1,000 kcal/day	108	85	99	119	132	0.52
Thiamin, mg/1,000 kcal/day	0.63	0.58	0.62	0.65	0.67	0.49
Riboflavin, mg/1,000 kcal/day	0.63	0.54	0.61	0.66	0.72	0.46
Niacin, mg/1,000 kcal/day	11.6	11.2	11.6	11.8	12.0	0.26
Vitamin B_6, mg/1,000 kcal/day	0.75	0.68	0.73	0.78	0.82	0.51
Vitamin B_{12}, mg/1,000 kcal/day	4.1	3.5	4.0	4.3	4.7	0.37
Pantothenic acid, mg/1,000 kcal/day	3.3	3.0	3.2	3.4	3.6	0.50
Folate, μg/1,000 kcal/day	196	164	185	212	226	0.52
Vitamin C, mg/1,000 kcal/day	65	50	58	72	78	0.56
Sodium, mg/1,000 kcal/day	1,251	1,059	1,253	1,297	1,402	0.29
Potassium, mg/1,000 kcal/day	1,377	1,171	1,320	1,460	1,575	0.55
Iron, mg/1,000 kcal/day	4.6	4.0	4.5	4.8	5.1	0.43
Calcium, mg/1,000 kcal/day	300	251	288	312	349	0.45
Magnesium, mg/1,000 kcal/day	175	165	175	177	185	0.35
Zinc, mg/1,000 kcal/day	4.8	4.6	4.8	4.8	4.9	0.32
Copper, mg/1,000 kcal/day	0.74	0.71	0.74	0.74	0.76	0.22
Manganese, mg/1,000 kcal/day	2.8	2.9	2.9	2.8	2.7	-0.19
Total dietary fiber, g/1,000 kcal/day	7.9	7.0	7.7	8.2	8.7	0.47
Soluble dietary fiber, g/1,000 kcal/day	1.7	1.6	1.7	1.8	1.9	0.45
Insoluble dietary fiber, g/1,000 kcal/day	5.7	5.1	5.6	5.9	6.3	0.46

DDS, dietary diversity score; MUFA, monounsaturated fatty acid; PUFA, polyunsaturated fatty acid; SFA; saturated fatty acid.

Four groups stratified by dietary diversity score (DDS) in baseline and 5 years later: Low/Low, low baseline DDS/low DDS in 5 year later; Low/High, low baseline DDS/high DDS in 5 year later; High/Low, high baseline DDS/low DDS in 5 year later; High/High, high baseline DDS/high DDS in 5 year later. Nutrient intake was adjusted for energy intake via the nutrient density method, using energy intake. Values are shown as medians in each group. MUFA, monounsaturated fatty acid; PUFA, polyunsaturated fatty acid; SFA, saturated fatty acid

[a] Spearman's correlation analysis was used to evaluate the relationship between nutrient intake and adherence score

[b] Sum of retinol, β-carotene/12, α-carotene/24, and cryptoxanthin/24

eTable 4. Association between food and beverage consumption and dietary diversity score change groups

	Total	Groups of dietary diversity score change				Baseline DDS
	(n=20,863)	Low/Low (n=7,866)	Low/High (n=2,951)	High/Low (n=3,000)	High/High (n=7,046)	*r* [a]
Cereals, g/1,000 kcal/day	297.9	331.8	318.7	283.1	269.1	-0.45
Potatoes, g/1,000 kcal/day	9.1	7.0	8.1	12.1	15.9	0.43
Sugar, g/1,000 kcal/day	1.8	1.7	1.5	1.6	2.0	0.00
Pulses, g/1,000 kcal/day	42.1	35.6	40.8	42.9	49.5	0.29
Vegetables, g/1,000 kcal/day	95.3	71.7	84.3	109.8	120.7	0.56
Fruits, g/1,000 kcal/day	78.8	53.6	64.4	98.1	104.2	0.44
Mushrooms, g/1,000 kcal/day	2.1	1.0	1.9	2.4	2.6	0.33
Seaweeds, g/1,000 kcal/day	3.1	2.0	2.5	3.9	4.3	0.45
Fish, g/1,000 kcal/day	25.2	19.5	22.8	28.3	30.4	0.33
Meat, g/1,000 kcal/day	16.6	13.5	15.4	18.3	19.8	0.31
Eggs, g/1,000 kcal/day	14.0	9.8	12.4	15.7	18.0	0.30
Dairy products, g/1,000 kcal/day	68.6	44.0	57.4	73.6	87.6	0.24
Fat and Oil, g/1,000 kcal/day	2.0	1.7	1.7	2.0	2.4	0.12
Confectioneries, g/1,000 kcal/day	5.5	4.1	5.2	5.9	7.2	0.24
Alcoholic beverages, g/1,000 kcal/day	0.0	0.0	0.0	0.0	0.0	-0.16
Non-alcoholic beverages, g/1,000 kcal/day	273.5	268.2	282.7	261.3	280.9	0.00
Seasonings, g/1,000 kcal/day	8.1	7.7	8.8	7.6	8.3	-0.03
Cooked foods, g/1,000 kcal/day	15.0	12.8	14.7	16.0	15.9	0.21

DDS, dietary diversity score.

Four groups stratified by dietary diversity score (DDS) in baseline and 5 years later: Low/Low, low baseline DDS/low DDS in 5 year later; Low/High, low baseline DDS/high DDS in 5 year later; High/Low, high baseline DDS/low DDS in 5 year later; High/High, high baseline DDS/high DDS in 5 year later. Dietary intake was adjusted for energy intake via the nutrient density method, using energy intake. Values are shown as medians in each group.

[a] Spearman's correlation analysis was used to evaluate the relationship between nutrient intake and adherence score

eTable 5. Association between energy and nutrient intake and longitudinal dietary diversity score change groups in men

	**Total** (n=7,719)	**Groups of dietary diversity score change**				**Baseline DDS**
		Low/Low (n=3,706)	Low/High (n=1,092)	High/Low (n=1,111)	High/High (n=1,810)	*r* [a]
Energy intake, kcal/day	1,766	1,636	1,705	1,945	1,980	0.36
Protein, % energy/day	12.3	11.3	12.2	13.1	13.6	0.52
Fat, % energy/day	16.3	14.5	15.7	17.9	18.8	0.49
Carbohydrate, % energy/day	57.9	59.0	58.6	56.5	56.8	-0.14
SFA, % energy/day	4.9	4.3	4.6	5.4	5.6	0.41
MUFA, % energy/day	5.1	4.5	4.9	5.8	6.0	0.48
PUFA, % energy/day	3.9	3.5	3.9	4.2	4.5	0.43
n-6 PUFA, % energy/day	3.2	2.8	3.1	3.4	3.6	0.39
n-3 PUFA, % energy/day	0.8	0.7	0.7	0.8	0.9	0.46
Cholesterol, mg/1,000 kcal/day	129	110	122	145	151	0.45
Water intake, g/1,000 kcal/day	739	697	737	771	803	0.21
Vitamin A, μg RE/1,000 kcal/day [b]	323	275	310	373	388	0.38
Vitamin D, μg/1,000 kcal/day	3.6	3.0	3.4	4.1	4.4	0.43
α-tocopherol, mg/1,000 kcal/day	2.8	2.5	2.7	3.1	3.2	0.61
Vitamin K, μg/1,000 kcal/day	91	75	87	109	116	0.54
Thiamin, mg/1,000 kcal/day	0.56	0.53	0.56	0.59	0.61	0.48
Riboflavin, mg/1,000 kcal/day	0.54	0.48	0.53	0.60	0.63	0.45
Niacin, mg/1,000 kcal/day	10.7	10.4	10.7	11.1	11.1	0.27
Vitamin B_6, mg/1,000 kcal/day	0.66	0.61	0.65	0.71	0.74	0.54
Vitamin B_{12}, mg/1,000 kcal/day	3.7	3.3	3.7	4.1	4.3	0.37
Pantothenic acid, mg/1,000 kcal/day	2.9	2.8	2.9	3.1	3.2	0.49
Folate, μg/1,000 kcal/day	169	147	164	191	201	0.54
Vitamin C, mg/1,000 kcal/day	51	42	48	60	65	0.58
Sodium, mg/1,000 kcal/day	1,167	1,012	1,190	1,251	1,323	0.28
Potassium, mg/1,000 kcal/day	1,157	1,030	1,128	1,277	1,350	0.55
Iron, mg/1,000 kcal/day	4.1	3.7	4.1	4.4	4.7	0.43
Calcium, mg/1,000 kcal/day	255	223	248	275	298	0.42
Magnesium, mg/1,000 kcal/day	159	152	159	164	169	0.34
Zinc, mg/1,000 kcal/day	4.4	4.3	4.4	4.5	4.6	0.30
Copper, mg/1,000 kcal/day	0.68	0.66	0.69	0.70	0.72	0.24
Manganese, mg/1,000 kcal/day	2.7	2.7	2.7	2.6	2.6	-0.12
Total dietary fiber, g/1,000 kcal/day	6.9	6.4	6.8	7.4	7.8	0.47
Soluble dietary fiber, g/1,000 kcal/day	1.5	1.4	1.5	1.6	1.7	0.46
Insoluble dietary fiber, g/1,000 kcal/day	5.0	4.6	5.0	5.4	5.6	0.46

DDS, dietary diversity score; MUFA, monounsaturated fatty acid; PUFA, polyunsaturated fatty acid; SFA; saturated fatty acid.

Four groups stratified by dietary diversity score (DDS) in baseline and 5 years later: Low/Low, low baseline DDS/low DDS in 5 year later; Low/High, low baseline DDS/high DDS in 5 year later; High/Low, high baseline DDS/low DDS in 5 year later; High/High, high baseline DDS/high DDS in 5 year later. Nutrient intake was adjusted for energy intake via the nutrient density method, using energy intake. Values are shown as medians in each group. MUFA, monounsaturated fatty acid; PUFA, polyunsaturated fatty acid; SFA, saturated fatty acid

[a] Spearman's correlation analysis was used to evaluate the relationship between nutrient intake and adherence score

[b] Sum of retinol, β-carotene/12, α-carotene/24, and cryptoxanthin/24

eTable 6. Association between energy and nutrient intake and longitudinal dietary diversity score change groups in women

	Total (n=13,144)	Groups of dietary diversity score change				Baseline DDS
		Low/Low (n=4,160)	Low/High (n=1,859)	High/Low (n=1,889)	High/High (n=5,236)	r [a]
Energy intake, kcal/day	1419	1247	1295	1500	1556	0.51
Protein, % energy/day	14.7	13.4	14.2	15.0	15.7	0.51
Fat, % energy/day	20.6	17.9	19.2	21.2	22.4	0.49
Carbohydrate, % energy/day	62.8	66.3	64.6	62.2	60.6	-0.43
SFA, % energy/day	6.3	5.4	5.8	6.5	6.9	0.40
MUFA, % energy/day	6.6	5.7	6.0	6.8	7.2	0.47
PUFA, % energy/day	4.7	4.2	4.6	4.8	5.1	0.40
n-6 PUFA, % energy/day	3.8	3.3	3.7	3.8	4.1	0.36
n-3 PUFA, % energy/day	0.9	0.8	0.9	1.0	1.0	0.40
Cholesterol, mg/1,000 kcal/day	158	128	141	167	183	0.43
Water intake, g/1,000 kcal/day	896	854	887	888	930	0.13
Vitamin A, μg RE/1,000 kcal/day [b]	394	305	354	406	458	0.36
Vitamin D, μg/1,000 kcal/day	4.4	3.7	4.0	4.9	5.1	0.38
α-tocopherol, mg/1,000 kcal/day	3.6	3.1	3.3	3.7	3.9	0.57
Vitamin K, μg/1,000 kcal/day	119	95	107	128	138	0.47
Thiamin, mg/1,000 kcal/day	0.66	0.62	0.64	0.67	0.69	0.46
Riboflavin, mg/1,000 kcal/day	0.69	0.59	0.64	0.70	0.75	0.42
Niacin, mg/1,000 kcal/day	12.1	11.8	12.0	12.2	12.3	0.17
Vitamin B_6 , mg/1,000 kcal/day	0.80	0.73	0.77	0.82	0.85	0.47
Vitamin B_{12}, mg/1,000 kcal/day	4.3	3.7	4.2	4.5	4.8	0.33
Pantothenic acid, mg/1,000 kcal/day	3.5	3.2	3.4	3.6	3.7	0.48
Folate, μg/1,000 kcal/day	211	180	197	222	234	0.48
Vitamin C, mg/1,000 kcal/day	73	59	65	78	82	0.51
Sodium, mg/1,000 kcal/day	1,308	1,098	1,321	1,325	1,437	0.25
Potassium, mg/1,000 kcal/day	1,495	1,297	1,410	1,542	1,629	0.53
Iron, mg/1,000 kcal/day	4.9	4.3	4.8	5.0	5.3	0.38
Calcium, mg/1,000 kcal/day	329	275	312	331	364	0.41
Magnesium, mg/1,000 kcal/day	183	174	183	184	189	0.28
Zinc, mg/1,000 kcal/day	4.9	4.8	4.9	4.9	5.0	0.27
Copper, mg/1,000 kcal/day	0.76	0.74	0.77	0.77	0.78	0.13
Manganese, mg/1,000 kcal/day	2.9	3.1	3.0	2.8	2.8	-0.30
Total dietary fiber, g/1,000 kcal/day	8.4	7.6	8.2	8.6	9.0	0.41
Soluble dietary fiber, g/1,000 kcal/day	1.9	1.7	1.8	1.9	2.0	0.39
Insoluble dietary fiber, g/1,000 kcal/day	6.1	5.5	5.9	6.2	6.4	0.40

DDS, dietary diversity score; MUFA, monounsaturated fatty acid; PUFA, polyunsaturated fatty acid; SFA; saturated fatty acid.

Four groups stratified by dietary diversity score (DDS) in baseline and 5 years later: Low/Low, low baseline DDS/low DDS in 5 year later; Low/High, low baseline DDS/high DDS in 5 year later; High/Low, high baseline DDS/low DDS in 5 year later; High/High, high baseline DDS/high DDS in 5 year later. Nutrient intake was adjusted for energy intake via the nutrient density method, using energy intake. Values are shown as medians in each group. MUFA, monounsaturated fatty acid; PUFA, polyunsaturated fatty acid; SFA, saturated fatty acid

[a] Spearman's correlation analysis was used to evaluate the relationship between nutrient intake and adherence score

[b] Sum of retinol, β-carotene/12, α-carotene/24, and cryptoxanthin/24

eTable 7. Association between food and beverage consumption and dietary diversity score change groups by sex

	Total	Groups of dietary diversity score change				Baseline DDS
	(n=20,863)	Low/Low (n=7,866)	Low/High (n=2,951)	High/Low (n=3,000)	High/High (n=7,046)	r [a]
**Men, n**	7,719	3,706	1,092	1,111	1,810	
Cereals, g/1,000 kcal/day	299.6	319.3	311.2	276.7	270.5	-0.33
Potatoes, g/1,000 kcal/day	6.4	5.1	6.2	9.1	11.0	0.42
Sugar, g/1,000 kcal/day	1.5	1.8	1.2	1.5	1.4	-0.05
Pulses, g/1,000 kcal/day	35.3	31.9	35.1	37.7	41.2	0.26
Vegetables, g/1,000 kcal/day	76.4	60.1	68.3	97.5	102.5	0.59
Fruits, g/1,000 kcal/day	56.4	40.5	46.9	76.6	81.3	0.44
Mushrooms, g/1,000 kcal/day	1.5	0.7	1.0	2.0	2.1	0.33
Seaweeds, g/1,000 kcal/day	2.4	1.7	2.1	3.3	3.7	0.47
Fish, g/1,000 kcal/day	21.6	18.0	20.4	26.2	27.7	0.34
Meat, g/1,000 kcal/day	14.0	11.9	13.5	16.5	16.9	0.31
Eggs, g/1,000 kcal/day	12.8	9.8	11.3	15.4	15.8	0.29
Dairy products, g/1,000 kcal/day	55.9	39.2	45.6	66.8	70.5	0.22
Fat and Oil, g/1,000 kcal/day	1.6	1.5	1.3	1.6	1.8	0.06
Confectioneries, g/1,000 kcal/day	3.7	1.6	3.6	4.2	5.4	0.24
Alcoholic beverages, g/1,000 kcal/day	101.8	116.3	103.4	97.9	81.8	-0.14
Non-alcoholic beverages, g/1,000 kcal/day	244.7	239.8	256.5	239.9	251.1	0.01
Seasonings, g/1,000 kcal/day	7.1	7.3	7.6	6.8	6.8	-0.07
Cooked foods, g/1,000 kcal/day	12.9	11.8	12.8	14.2	14.5	0.23
**Women, n**	13,144	4,160	1,859	1,889	5,236	
Cereals, g/1,000 kcal/day	297.2	340.6	321.7	286.1	268.9	-0.54
Potatoes, g/1,000 kcal/day	11.5	8.6	9.0	14.6	17.2	0.37
Sugar, g/1,000 kcal/day	2.0	1.6	1.7	1.7	2.2	0.01
Pulses, g/1,000 kcal/day	46.9	39.7	44.8	46.9	52.9	0.25
Vegetables, g/1,000 kcal/day	107.2	82.5	93.0	119.6	127.4	0.50
Fruits, g/1,000 kcal/day	94.1	67.3	74.0	111.6	111.6	0.38
Mushrooms, g/1,000 kcal/day	2.5	2.0	2.4	2.6	2.7	0.26
Seaweeds, g/1,000 kcal/day	3.6	2.4	2.8	4.2	4.6	0.40
Fish, g/1,000 kcal/day	27.3	21.9	24.3	29.3	31.5	0.29
Meat, g/1,000 kcal/day	18.3	15.4	16.6	19.3	20.8	0.27
Eggs, g/1,000 kcal/day	14.8	9.7	13.0	16.6	19.3	0.28
Dairy products, g/1,000 kcal/day	80.1	49.6	62.8	81.5	93.6	0.21
Fat and Oil, g/1,000 kcal/day	2.3	1.9	2.0	2.2	2.6	0.12
Confectioneries, g/1,000 kcal/day	6.4	5.8	6.0	7.1	8.4	0.18
Alcoholic beverages, g/1,000 kcal/day	0.0	0.0	0.0	0.0	0.0	-0.02
Non-alcoholic beverages, g/1,000 kcal/day	294.0	299.6	302.7	277.1	293.3	-0.03
Seasonings, g/1,000 kcal/day	8.8	8.5	10.0	8.4	8.8	-0.09
Cooked foods, g/1,000 kcal/day	16.0	14.7	16.1	16.4	16.1	0.15

DDS, dietary diversity score.

Four groups stratified by dietary diversity score (DDS) in baseline and 5 years later: Low/Low, low baseline DDS/low DDS in 5 year later; Low/High, low baseline DDS/high DDS in 5 year later; High/Low, high baseline DDS/low DDS in 5 year later; High/High, high baseline DDS/high DDS in 5 year later. Dietary intake was adjusted for energy intake via the nutrient density method, using energy intake. Values are shown as medians in each group.

[a] Spearman's correlation analysis was used to evaluate the relationship between nutrient intake and adherence score

eTable 8. Association between dietary diversity score for each food group and dietary diversity score change groups by sex

	Total		Groups of dietary diversity score change							
			Low/Low		Low/High		High/Low		High/High	
Women, n	13,144		4,160		1,859		1,889		5,236	
Men, n	7,719		3,706		1,092		1,111		1,810	
**Baseline**	Mean	(%)	Mean	(%)	Mean	(%)	Mean	(%)	Mean	(%)
Total DDS										
Women	11.0	(100)	7.6	(100)	8.6	(100)	12.7	(100)	14.0	(100)
Men	9.7	(100)	7.1	(100)	8.3	(100)	12.7	(100)	13.8	(100)
Meat										
Women	0.78	(7.1)	0.56	(7.3)	0.62	(7.2)	0.89	(7.0)	0.97	(6.9)
Men	0.75	(7.8)	0.58	(8.2)	0.66	(8.0)	0.99	(7.8)	1.01	(7.3)
Fish										
Women	0.94	(8.5)	0.69	(9.1)	0.76	(8.8)	1.10	(8.6)	1.15	(8.2)
Men	0.93	(9.6)	0.73	(10.3)	0.82	(9.9)	1.18	(9.3)	1.23	(8.3)
Dairy										
Women	0.76	(6.9)	0.49	(6.5)	0.61	(7.0)	0.81	(6.3)	1.01	(7.2)
Men	0.65	(6.8)	0.48	(6.7)	0.58	(7.0)	0.84	(6.6)	0.94	(6.8)
Soy										
Women	0.73	(6.6)	0.50	(6.5)	0.58	(6.7)	0.81	(6.4)	0.95	(6.8)
Men	0.64	(6.7)	0.48	(6.7)	0.57	(6.8)	0.81	(6.4)	0.92	(6.7)
Vegetables and fruits										
Women	4.24	(38.4)	2.92	(38.4)	3.20	(37.1)	5.03	(39.5)	5.38	(38.4)
Men	3.62	(37.5)	2.60	(36.6)	3.00	(36.2)	4.92	(38.6)	5.27	(38.2)
Other										
Women	3.07	(27.8)	2.05	(26.9)	2.21	(25.6)	3.67	(28.8)	3.98	(28.4)
Men	2.67	(27.6)	1.94	(27.3)	2.18	(26.3)	3.58	(28.1)	3.89	(28.2)
**5 years later**										
Total DDS										
Women	10.8	(100)	7.6	(100)	12.3	(100)	8.5	(100)	13.7	(100)
Men	9.5	(100)	7.1	(100)	12.4	(100)	8.1	(100)	13.5	(100)
Meat										
Women	0.72	(6.6)	0.53	(6.9)	0.81	(6.6)	0.57	(6.7)	0.88	(6.5)
Men	0.70	(7.3)	0.56	(7.8)	0.85	(6.9)	0.62	(7.7)	0.94	(6.9)
Fish										
Women	0.91	(8.4)	0.69	(9.1)	1.04	(8.4)	0.72	(8.5)	1.12	(8.2)
Men	0.90	(9.5)	0.72	(10.1)	1.17	(9.4)	0.76	(9.4)	1.21	(8.9)
Dairy										
Women	0.89	(8.3)	0.61	(8.0)	0.98	(7.9)	0.71	(8.4)	1.16	(8.5)
Men	0.72	(7.6)	0.53	(7.4)	0.92	(7.4)	0.67	(8.3)	1.03	(7.6)
Soy										
Women	0.75	(6.9)	0.53	(7.0)	0.85	(6.9)	0.59	(6.9)	0.95	(7.0)
Men	0.67	(7.1)	0.51	(7.2)	0.88	(7.1)	0.55	(6.8)	0.96	(7.1)
Vegetables and fruits										
Women	4.34	(40.1)	3.05	(40.1)	4.99	(40.5)	3.39	(40.0)	5.47	(40.0)
Men	3.72	(39.1)	2.73	(38.5)	4.95	(39.9)	3.15	(39.0)	5.34	(39.5)
Other										
Women	3.01	(27.8)	2.06	(27.1)	3.42	(27.7)	2.32	(27.3)	3.86	(28.2)
Men	2.62	(27.6)	1.93	(27.2)	3.47	(28.0)	2.16	(26.7)	3.82	(28.3)

DDS, dietary diversity score.

The food items were categorized as follows: meat (beef, pork, ham or sausage, chicken, and liver); fish (fresh fish, kamaboko, and dried or salted fish); dairy products (milk, yogurt, and cheese); soy (boiled beans and tofu); vegetables and fruits (fried vegetables, spinach or garland chrysanthemum, carrots or pumpkins, tomatoes, cabbage or head lettuce, Chinese cabbage, edible wild plants, pickles, citrus, and non-citrus fruits).

eTable 9. Sensitivity analysis results for the relationship between longitudinal longitudinal dietary diversity score change groups and all-cause and cause-specific mortality after excluding participants with an event in the first 5 years of follow-up

	**Groups of dietary diversity score change**							
	Low/Low (n=7,543)		Low/High (n=2,832)		High/Low (n=2,886)		High/High (n=6,860)	
Person years	94,201		36,285		36,653		87,007	
Baseline DDS [a]	7.4	(2.0)	8.5	(1.6)	12.7	(2.2)	14.0	(2.6)
5 years later DDS [a]	7.4	(1.9)	12.4	(1.9)	8.3	(1.6)	13.6	(2.5)
Total mortality								
Number of deaths	944		363		317		629	
Rate/1,000 PY (95% CI)	10.0	(9.4–10.7)	10.0	(9.0–11.1)	8.6	(7.7–9.7)	7.2	(6.7–7.8)
Model 1 [b]	1.00	(Ref)	0.98	(0.87–1.11)	0.89	(0.78–1.01)	0.78	(0.70–0.86)
Model 2 [c]	1.00	(Ref)	1.00	(0.88–1.13)	0.93	(0.82–1.07)	0.84	(0.75–0.95)
Cancer mortality								
Number of deaths	334		115		121		269	
Rate/1,000 PY (95% CI)	3.5	(3.2–3.9)	3.2	(2.6–3.8)	3.3	(2.8–3.9)	3.1	(2.7–3.5)
Model 1 [b]	1.00	(Ref)	0.91	(0.74–1.13)	0.97	(0.79–1.20)	0.98	(0.83–1.16)
Model 2 [c]	1.00	(Ref)	0.92	(0.74–1.14)	0.98	(0.79–1.22)	1.02	(0.85–1.22)
CVD mortality								
Number of deaths	258		101		80		159	
Rate/1,000 PY (95% CI)	2.7	(2.4–3.1)	2.8	(2.3–3.4)	2.2	(1.8–2.7)	1.8	(1.6–2.1)
Model 1 [b]	1.00	(Ref)	0.98	(0.78–1.24)	0.79	(0.62–1.02)	0.69	(0.56–0.85)
Model 2 [c]	1.00	(Ref)	1.00	(0.79–1.27)	0.89	(0.68–1.15)	0.79	(0.63–0.98)
RD mortality								
Number of deaths	111		51		41		79	
Rate/1,000 PY (95% CI)	1.2	(1.0–1.4)	1.4	(1.1–1.8)	1.1	(0.8–1.5)	0.9	(0.7–1.1)
Model 1 [b]	1.00	(Ref)	1.16	(0.83–1.63)	1.02	(0.71–1.46)	0.87	(0.64–1.17)
Model 2 [c]	1.00	(Ref)	1.25	(0.89–1.76)	1.15	(0.79–1.68)	0.98	(0.71–1.35)

CI, confidence interval; CVD, cardiovascular disease; DDS, dietary diversity score; PY, person-years; RD, respiratory disease; Ref, reference.

Four groups stratified by dietary diversity score in baseline and 5 years later: Low/Low, low baseline DDS/low DDS in 5 year later; Low/High, low baseline DDS/high DDS in 5 year later; High/Low, high baseline DDS/low DDS in 5 year later; High/High, high baseline DDS/high DDS in 5 year later.

[a] Dietary diversity scores are shown as means and standard deviations.

[b] Model 1: Adjusted for baseline age, baseline areas, and baseline sex.

[c] Model 2: In addition to the factors listed in model 1, adjusted for baseline body mass index, 5 years later smoking status, 5 years later alcohol drinking, baseline occupation status, baseline educational attainment, baseline marital status, baseline energy intake, baseline green tea consumption, baseline coffee consumption, baseline time spent watching television, baseline sleep duration, baseline sports or exercise status, baseline walking status, 5 years later history of diabetes, and baseline history of hypertension.

eTable 10. Sensitivity analysis results for the relationship between dietary diversity score change groups and all-cause and cause-specific mortality using a multiple imputation method for missing value of covariates

	**Groups of dietary diversity score change**							
	Low/Low (n=7,866)		Low/High (n=2,951)		High/Low (n=3,000)		High/High (n=7,046)	
Person years	95,143		36,616		36,982		87,536	
Baseline DDS [a]	7.4	(2.0)	8.5	(1.6)	12.7	(2.2)	14.0	(2.6)
5 years later DDS [a]	7.4	(1.9)	12.4	(1.9)	8.3	(1.6)	13.6	(2.5)
Total mortality								
Number of deaths	1267		482		431		815	
Rate/1,000 PY (95% CI)	13.3	(12.6–14.1)	13.2	(12.0–14.4)	11.7	(10.6–12.8)	9.3	(8.7–10.0)
Model 1 [b]	1.00	(Ref)	0.99	(0.89–1.10)	0.91	(0.82–1.02)	0.76	(0.70–0.84)
Model 2 [c]	1.00	(Ref)	1.00	(0.90–1.12)	0.95	(0.85–1.07)	0.82	(0.74–0.91)
Cancer mortality								
Number of deaths	472		170		177		350	
Rate/1,000 PY (95% CI)	5.0	(4.5–5.4)	4.6	(4.0–5.4)	4.8	(4.1–5.5)	4.0	(3.6–4.4)
Model 1 [b]	1.00	(Ref)	0.97	(0.81–1.16)	1.03	(0.86–1.22)	0.92	(0.80–1.07)
Model 2 [c]	1.00	(Ref)	0.96	(0.80–1.15)	1.03	(0.86–1.23)	0.93	(0.80–1.09)
CVD mortality								
Number of deaths	345		134		101		213	
Rate/1,000 PY (95% CI)	3.6	(3.3–4.0)	3.7	(3.1–4.3)	2.7	(2.2–3.3)	2.4	(2.1–2.8)
Model 1 [b]	1.00	(Ref)	0.98	(0.80–1.20)	0.76	(0.61–0.95)	0.70	(0.59–0.84)
Model 2 [c]	1.00	(Ref)	1.01	(0.83–1.24)	0.85	(0.67–1.07)	0.81	(0.67–0.98)
RD mortality								
Number of deaths	135		60		50		91	
Rate/1,000 PY (95% CI)	1.4	(1.2–1.7)	1.6	(1.3–2.1)	1.4	(1.0–1.8)	1.0	(0.8–1.3)
Model 1 [b]	1.00	(Ref)	1.13	(0.83–1.54)	1.03	(0.74–1.43)	0.82	(0.63–1.08)
Model 2 [c]	1.00	(Ref)	1.22	(0.89–1.67)	1.12	(0.80–1.58)	0.93	(0.69–1.26)

CI, confidence interval; CVD, cardiovascular disease; DDS, dietary diversity score; PY, person-years; RD, respiratory disease; Ref, reference.

[a] Dietary diversity scores are shown as means and standard deviations.

[b] Model 1: Adjusted for baseline age, baseline areas, and baseline sex.

[c] Model 2: Adjusted for baseline body mass index, 5 years later smoking status, 5 years later alcohol drinking, baseline occupation status, baseline educational attainment, baseline marital status, baseline energy intake, baseline green tea consumption, baseline coffee consumption, baseline time spent watching television, baseline sleep duration, baseline sports or exercise status, baseline walking status, 5 years later history of diabetes, and baseline history of hypertension.

eTable 11. Sensitivity analysis results for the relationship between dietary diversity score change groups and all-cause and cause-specific mortality using uniform dietary diversity cutoff values at both baseline and 5 years later

	**Groups of dietary diversity score change**							
	Low/Low (n=8,073)		Low/High (n=2,744)		High/Low (n=3,216)		High/High (n=6,830)	
Person years	97831		33928		39780		84739	
Baseline DDS [a]	7.4	(2.0)	8.5	(1.6)	12.8	(2.2)	14.0	(2.6)
DDS in 5 years [a]	7.4	(1.9)	12.5	(1.9)	8.5	(1.6)	13.7	(2.5)
Total mortality								
Number of deaths	1295		454		454		792	
Rate/1,000 PY (95% CI)	13.2	(12.5–14.0)	13.4	(12.2–14.7)	11.4	(10.4–12.5)	9.3	(8.7–10.0)
Model 1 [b]	1.00	(Ref)	1.01	(0.90–1.12)	0.89	(0.80–1.00)	0.77	(0.71–0.85)
Model 2 [c]	1.00	(Ref)	1.02	(0.91–1.14)	0.91	(0.82–1.02)	0.81	(0.74–0.89)
Cancer mortality								
Number of deaths	478		164		188		339	
Rate/1,000 PY (95% CI)	4.9	(4.5–5.3)	4.8	(4.1–5.6)	4.7	(4.1–5.5)	4.0	(3.6–4.4)
Model 1 [b]	1.00	(Ref)	1.02	(0.85–1.22)	1.03	(0.87–1.22)	0.94	(0.81–1.09)
Model 2 [c]	1.00	(Ref)	1.02	(0.85–1.22)	1.03	(0.86–1.22)	0.96	(0.83–1.11)
CVD mortality								
Number of deaths	356		123		104		210	
Rate/1,000 PY (95% CI)	3.6	(3.3–4.0)	3.6	(3.0–4.3)	2.6	(2.2–3.2)	2.5	(2.2–2.8)
Model 1 [b]	1.00	(Ref)	0.97	(0.78–1.19)	0.72	(0.58–0.90)	0.72	(0.60–0.85)
Model 2 [c]	1.00	(Ref)	0.98	(0.79–1.20)	0.76	(0.61–0.95)	0.76	(0.64–0.92)
RD mortality								
Number of deaths	137		58		52		89	
Rate/1,000 PY (95% CI)	1.4	(1.2–1.7)	1.7	(1.3–2.2)	1.3	(1.0–1.7)	1.1	(0.9–1.3)
Model 1 [b]	1.00	(Ref)	1.18	(0.86–1.61)	1.00	(0.72–1.38)	0.85	(0.64–1.12)
Model 2 [c]	1.00	(Ref)	1.27	(0.92–1.74)	1.04	(0.75–1.44)	0.92	(0.69–1.22)

CI, confidence interval; CVD, cardiovascular disease; DDS, dietary diversity score; PY, person-years; RD, respiratory disease; Ref, reference.

[a] Dietary diversity scores are shown as means and standard deviations.

[b] Model 1: Adjusted for baseline age, baseline areas, and baseline sex.

[c] Model 2: Adjusted for baseline body mass index, 5 years later smoking status, 5 years later alcohol drinking, baseline occupation status, baseline educational attainment, baseline marital status, baseline energy intake, baseline green tea consumption, baseline coffee consumption, baseline time spent watching television, baseline sleep duration, baseline sports or exercise status, baseline walking status, 5 years later history of diabetes, and baseline history of hypertension.

eTable 12. Hazard ratios for baseline and 5 years later dietary diversity score and all-cause and cause-specific mortality calculated using a multivariate Cox proportional hazards model

	**Baseline survey**				**Additional survey**			
	Low DDS (n=10,817)		High DDS (n=10,046)		Low DDS (n=10,866)		High DDS (n=9,997)	
Person years	131,759		124,518		132,125		124,152	
Total mortality								
Number of deaths	1,749		1,246		1,698		1,297	
Rate/1,000 PY (95% CI)	13.3	(12.7–13.9)	10.0	(9.5–10.6)	12.9	(12.3–13.5)	10.4	(9.9–11.0)
Model 1 [a]	1.00	(Ref)	0.81	(0.76–0.88)	1.00	(Ref)	0.86	(0.80–0.93)
Model 2 [b]	1.00	(Ref)	0.87	(0.80–0.94)	1.00	(Ref)	0.90	(0.84–0.98)
Cancer mortality								
Number of deaths	642		527		649		520	
Rate/1,000 PY (95% CI)	4.9	(4.5–5.3)	4.2	(3.9–4.6)	4.9	(4.5–5.3)	4.2	(3.8–4.6)
Model 1 [a]	1.00	(Ref)	0.97	(0.86–1.09)	1.00	(Ref)	0.93	(0.83–1.05)
Model 2 [b]	1.00	(Ref)	0.97	(0.85–1.10)	1.00	(Ref)	0.94	(0.83–1.06)
CVD mortality								
Number of deaths	479		314		446		347	
Rate/1,000 PY (95% CI)	3.6	(3.3–4.0)	2.5	(2.3–2.8)	3.4	(3.1–3.7)	2.8	(2.5–3.1)
Model 1 [a]	1.00	(Ref)	0.73	(0.63–0.84)	1.00	(Ref)	0.85	(0.74–0.98)
Model 2 [b]	1.00	(Ref)	0.82	(0.70–0.96)	1.00	(Ref)	0.93	(0.80–1.08)
RD mortality								
Number of deaths	195		141		185		151	
Rate/1,000 PY (95% CI)	1.5	(1.3–1.7)	1.1	(1.0–1.3)	1.4	(1.2–1.6)	1.2	(1.0–1.4)
Model 1 [a]	1.00	(Ref)	0.86	(0.69–1.07)	1.00	(Ref)	0.92	(0.74–1.14)
Model 2 [b]	1.00	(Ref)	0.94	(0.74–1.19)	1.00	(Ref)	0.99	(0.79–1.25)

CI, confidence interval; CVD, cardiovascular disease; DDS, dietary diversity score; PY, person-years; RD, respiratory disease; Ref, reference.

[a] Model 1: Adjusted for baseline age, baseline areas, and baseline sex.

[b] Model 2: Adjusted for body mass index, smoking status, alcohol drinking, occupation status, educational attainment, marital status, energy intake, green tea consumption, coffee consumption, time spent watching television, sleep duration, sports or exercise status, walking status, history of diabetes, and history of hypertension.

eTable 13. Association between change in dietary diversity score from baseline to 5 years later and all-cause and cause-specific mortality using a multivariate linear model among adults

	**Quartile of dietary diversity score change**							
	Q1 (n=5,355)		Q2 (n=5,297)		Q3 (n=5,065)		Q4 (n=5,146)	
Person years	66,318		64,459		61,601		63,900	
DDS change [a]	-4.3	(2.2)	-1.0	(0.6)	0.9	(0.5)	3.9	(2.0)
Total mortality								
Number of deaths	804		691		692		808	
Rate/1,000 PY (95% CI)	12.1	(11.3–13.0)	10.7	(9.9–11.5)	11.2	(10.4–12.1)	12.6	(11.8–13.5)
Model 1 [b]	0.99	(0.89–1.09)	0.93	(0.84–1.04)	1.00	(Ref)	1.03	(0.93–1.14)
Model 2 [c]	1.00	(0.90–1.11)	0.95	(0.85–1.05)	1.00	(Ref)	1.00	(0.90–1.11)
Cancer mortality								
Number of deaths	310		283		271		305	
Rate/1,000 PY (95% CI)	4.7	(4.2–5.2)	4.4	(3.9–4.9)	4.4	(3.9–5.0)	4.8	(4.3–5.3)
Model 1 [b]	0.99	(0.84–1.17)	0.98	(0.83–1.15)	1.00	(Ref)	1.01	(0.85–1.18)
Model 2 [c]	0.98	(0.83–1.16)	0.97	(0.82–1.15)	1.00	(Ref)	0.99	(0.84–1.17)
CVD mortality								
Number of deaths	206		180		196		211	
Rate/1,000 PY (95% CI)	3.1	(2.7–3.6)	2.8	(2.4–3.2)	3.2	(2.8–3.7)	3.3	(2.9–3.8)
Model 1 [b]	0.87	(0.72–1.06)	0.86	(0.70–1.05)	1.00	(Ref)	0.93	(0.77–1.13)
Model 2 [c]	0.92	(0.75–1.12)	0.90	(0.73–1.10)	1.00	(Ref)	0.90	(0.74–1.10)
RD mortality								
Number of deaths	83		81		69		103	
Rate/1,000 PY (95% CI)	1.3	(1.0–1.6)	1.3	(1.0–1.6)	1.1	(0.9–1.4)	1.6	(1.3–2.0)
Model 1 [b]	0.96	(0.70–1.33)	1.06	(0.77–1.46)	1.00	(Ref)	1.25	(0.92–1.70)
Model 2 [c]	0.95	(0.69–1.32)	1.07	(0.77–1.48)	1.00	(Ref)	1.24	(0.91–1.69)

CI, confidence interval; CVD, cardiovascular disease; DDS, dietary diversity score; PY, person-years; Q, quartile; RD, respiratory disease; Ref, reference.

[a] Dietary diversity scores are shown as means and standard deviations.

[b] Model 1: Adjusted for baseline age, baseline areas, and baseline sex.

[c] Model 2: Adjusted for body mass index, smoking status, alcohol drinking, occupation status, educational attainment, marital status, energy intake, green tea consumption, coffee consumption, time spent watching television, sleep duration, sports or exercise status, walking status, history of diabetes, and history of hypertension.

eTable 14. Hazard ratios for longitudinal dietary diversity score change groups and all-cause mortality calculated using sex and marital status stratified multivariate Cox proportional hazards models

	**Groups of dietary diversity score change**							
	Low/Low		Low/High		High/Low		High/High	
**Married men, n**	3,357		996		1,022		1,668	
Baseline DDS [a]	7.2	(2.0)	8.3	(1.6)	12.7	(2.2)	13.8	(2.6)
DDS in 5 years [a]	7.1	(1.9)	12.4	(1.9)	8.1	(1.7)	13.5	(2.5)
Person years	40,861		12,098		12,900		20,317	
Number of total deaths	663		240		203		353	
Rate/1,000 PY (95% CI)	16.2	(15.0–17.5)	19.8	(17.5–22.5)	15.7	(13.7–18.1)	17.4	(15.7–19.3)
Model 2 [b]	1.00	(Ref)	1.09	(0.93–1.26)	1.01	(0.86–1.19)	0.88	(0.76–1.02)
**Married women, n**	3311		1524		1537		4441	
Baseline DDS [a]	7.6	(1.8)	8.7	(1.5)	12.7	(2.1)	14.0	(2.6)
DDS in 5 years [a]	7.7	(1.8)	12.3	(1.8)	8.5	(1.5)	13.6	(2.5)
Person years	40,982		19,467		19,006		56,429	
Number of total deaths	343		144		146		321	
Rate/1,000 PY (95% CI)	8.4	(7.5–9.3)	7.4	(6.3–8.7)	7.7	(6.5–9.0)	5.7	(5.1–6.3)
Model 2 [b]	1.00	(Ref)	0.89	(0.73–1.09)	0.95	(0.77–1.17)	0.77	(0.65–0.92)
**Other men, n**	200		42		39		54	
Baseline DDS [a]	6.2	(2.1)	7.7	(2.1)	12.6	(1.6)	14.0	(2.5)
DDS in 5 years [a]	6.3	(2.1)	12.5	(2.1)	7.8	(1.9)	13.7	(2.3)
Person years	2,169		435		456		645	
Number of total deaths	53		18		11		16	
Rate/1,000 PY (95% CI)	24.4	(18.7–32.0)	41.4	(26.1–65.7)	24.1	(13.4–43.5)	24.8	(15.2–40.5)
Model 2 [b]	1.00	(Ref)	1.60	(0.79–3.21)	1.15	(0.47–2.82)	0.99	(0.44–2.27)
**Other women, n**	639		233		250		519	
Baseline DDS [a]	7.5	(2.0)	8.3	(1.7)	12.8	(2.2)	13.9	(2.6)
DDS in 5 years [a]	7.4	(1.8)	12.4	(1.9)	8.4	(1.5)	13.7	(2.5)
Person years	6,743		2,746		2,738		5,659	
Number of total deaths	123		43		48		67	
Rate/1,000 PY (95% CI)	18.2	(15.3–21.8)	15.7	(11.6–21.1)	17.5	(13.2–23.3)	11.8	(9.3–15.0)
Model 2 [b]	1.00	(Ref)	0.84	(0.57–1.22)	0.88	(0.60–1.28)	0.71	(0.50–1.01)

CI, confidence interval; DDS, dietary diversity score; HR, hazard ratio; PY, person-years; Ref, reference.

Other groups included divorced, single, and widowed status.

[a] Dietary diversity scores are shown as means and standard deviations.

[b] Model 2: Adjusted for baseline age, baseline areas, baseline sex, baseline body mass index, 5 years later smoking status, 5 years later alcohol drinking, baseline occupation status, baseline educational attainment, baseline marital status, baseline energy intake, baseline green tea consumption, baseline coffee consumption, baseline time spent watching television, baseline sleep duration, baseline sports or exercise status, baseline walking status, 5 years later history of diabetes, and baseline history of hypertension.

eTable 15. Sensitivity analysis results for the relationship between longitudinal dietary diversity score change groups and all-cause and cardiovascular disease mortality using the sex-stratified model after excluding participants with a history of diabetes and hypertension

	Groups of dietary diversity score change							
	Low/Low		Low/High		High/Low		High/High	
**Women, n**	2,989		1,362		1,389		3,995	
Person years	37,833		17,900		17,607		51,948	
Baseline DDS [a]	7.6	(1.9)	8.6	(1.5)	12.8	(2.2)	14.1	(2.7)
DDS in 5 years [a]	7.7	(1.8)	12.3	(1.8)	8.5	(1.4)	13.7	(2.5)
Total mortality								
Number of deaths	315		120		122		269	
Rate/1,000 PY (95% CI)	8.3	(7.5–9.3)	6.7	(5.6–8.0)	6.9	(5.8–8.3)	5.2	(4.6–5.8)
Model 2 [b]	1.00	(Ref)	0.82	(0.66–1.01)	0.85	(0.68–1.06)	0.73	(0.60–0.89)
CVD mortality								
Number of deaths	84		25		27		68	
Rate/1,000 PY (95% CI)	2.2	(1.8–2.7)	1.4	(0.9–2.1)	1.5	(1.1–2.2)	1.3	(1.0–1.7)
Model 2 [b]	1.00	(Ref)	0.67	(0.42–1.06)	0.80	(0.50–1.28)	0.68	(0.41–0.98)
**Men, n**	2,726		769		847		1,347	
Person years	34,115		9,647		11,003		16,818	
Baseline DDS [a]	7.1	(2.0)	8.3	(1.7)	12.8	(2.3)	13.8	(2.6)
DDS in 5 years [a]	7.1	(1.9)	12.4	(2.1)	8.0	(1.7)	13.5	(2.5)
Total mortality								
Number of deaths	498		176		156		281	
Rate/1,000 PY (95% CI)	14.6	(13.4–15.9)	18.2	(15.7–21.1)	14.2	(12.1–16.6)	16.7	(14.9–18.8)
Model 2 [b]	1.00	(Ref)	1.16	(0.97–1.38)	1.03	(0.85–1.24)	0.99	(0.84–1.16)
CVD mortality								
Number of deaths	113		38		30		54	
Rate/1,000 PY (95% CI)	3.3	(2.8–4.0)	3.9	(2.9–5.4)	2.7	(1.9–3.9)	3.2	(2.5–4.2)
Model 2 [b]	1.00	(Ref)	1.07	(0.73–1.57)	0.87	(0.57–1.33)	0.80	(0.56–1.15)

CI, confidence interval; CVD, cardiovascular disease; DDS, dietary diversity score; PY, person-years; Ref, reference.

[a] Dietary diversity scores are shown as means and standard deviations.

[b] Model 2: Adjusted for baseline age, baseline areas, baseline sex, baseline body mass index, 5 years later smoking status, 5 years later alcohol drinking, baseline occupation status, baseline educational attainment, baseline marital status, baseline energy intake, baseline green tea consumption, baseline coffee consumption, baseline time spent watching television, baseline sleep duration, baseline sports or exercise status, and baseline walking status.
